# Benchmark Dataset for Evaluation of Range-Based People Tracker Classifiers in Mobile Robots

**DOI:** 10.3389/fnbot.2017.00072

**Published:** 2018-01-15

**Authors:** Claudia Álvarez-Aparicio, Ángel Manuel Guerrero-Higueras, Maria Carmen Calvo Olivera, Francisco J. Rodríguez-Lera, Francisco Martín, Vicente Matellán

**Affiliations:** ^1^Grupo de Robótica, Universidad de León, León, Spain; ^2^University of Luxembourg, Luxembourg, Luxembourg; ^3^Robotics Lab, Universidad Rey Juan Carlos, Madrid, Spain

**Keywords:** people tracking, mobile robots, range-based location, LIDAR sensors, convolutional neural networks

## 1 Introduction

Detecting and tracking people is a very useful skill for different systems, in particular, for improving navigation social for mobile robots, or to facilitate more socially acceptable robots. Many solutions in the literature try to solve this problem using a multimodal approach, typically vision and range sensors, as seen in Arras et al. (2012). Vision sensors are more expensive and are more likely to gather contradictory information. For this reason, systems based only on range sensors are desirable. Regarding the classifiers for processing sensor data, convolutional neural networks are becoming a very popular solution, according to Long et al. (2015).

Laser imaging detection and ranging (LIDAR) sensors are reliable and currently affordable range sensors that provide information about the environment at good rates (~20−30 Hz.) for dynamic environments. They are easy to process on-board in real-time because each scan consists an array of only a few 100 integers.

Usually mobile robots mount laser scanners in a low position (~30−50 cm from the ground) to detect dynamic obstacles. They are also used to construct occupancy maps and navigate. The information provided allows estimating the distance at precise angles (resolution of 0.5°). This means that objects such as table or chair legs, trunks of plants, etc., may be easily confused with legs of persons. It is also difficult to keep track of a particular person (i.e., a pair of legs) in a crowded environment because many obstructions can result.

Fitting neural networks requires a good training dataset. Collecting and organizing a training set requires time as well as domain-specific knowledge. There is a large collection of robotic datasets available from various mobile robots, vehicles, and handheld sensors, such as Repository of robotics and computer vision datasets^1^ for Mobile Robot Programming Toolkit (MRPT). However, most datasets may not be suitable for training neural networks. This data report summarizes a benchmark dataset, which can be used to evaluate the performance of different approaches for detecting and tracking people by using LIDAR sensors. Information contained in the dataset is specially suitable for use as training data for neural network-based classifiers.

Data actually contained in the dataset allow evaluating two people trackers, both neural network-based: leg detector (LD), a widely used solution by the Robot Operating System (ROS) community, see Quigley et al. (2009); and a people-tracker tool developed by the Robotics Group at the University of Leon, known as PeTra.

The rest of this paper is organized as follows: Section 2 describes the systems and the environment where data were gathered. It also specifies the procedure and tools employed. Section 3 explains how the dataset can be accessed and enumerates some applications and limitations for the data included in the dataset.

## 2 Materials and Methods

**Figure 1:**
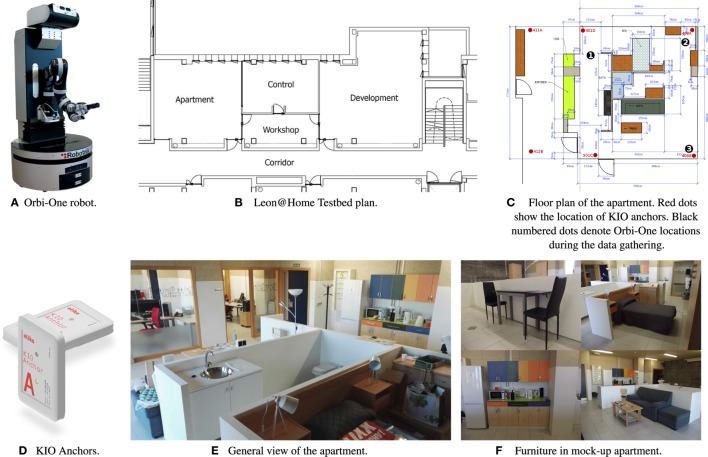
Materials used in the experiments. **(A)** Orbi-One robot. **(B)** Leon@Home Testbed plan. **(C)** Floor plan of the apartment. Red dots show the location of KIO anchors. Black numbered dots denote Orbi-One locations during the data gathering. **(D)** KIO anchors. **(E)** General view of the apartment. **(F)** Furniture in mock-up apartment.

The following section describes the materials (shown in Figure 1) used to gather data, which include: a certified study area, an autonomous robot with an on-board LIDAR sensor, and a real-time location system (RTLS) to obtain ground-truth data about person location. Recorded data include location estimates calculated by two people trackers, LD and PeTra, also described below. Finally, the recording procedure used to build the dataset is explained.

### 2.1 Leon@Home Testbed

Data have been gathered at Leon@Home Testbed.^2^ This is a Certified Testbed^3^ of the European Robotics league (ERL). Its main purpose is to benchmark service robots in a realistic home environment. Our testbed is made up of four parts, shown in Figure 1B: a mock-up apartment, a control zone with direct vision (glass wall) into the apartment, a small workshop, and a larger development zone, where researchers work.

Leon@Home Testbed is located on the second floor of the Módulo de Investigación en Cibernética (Building for Research in Cybernetics) on the Vegazana Campus of the University of León (Spain). The apartment is a single bedroom mock-up home built in an 8 m × 7 m space. Figure 1C shows a plan of the apartment. 60 cm high walls divide it into a kitchen, living room, bathroom, and bedroom. The furniture (Figures 1E,F) has been chosen to test different robot abilities. For instance, the kitchen cabinets all have different types of handles.

### 2.2 Orbi-One Robot

Orbi-One (Figure 1A) is an assistant robot manufactured by Robotnik.^4^ It has several sensors, among them, a RGBD camera, a LIDAR sensor, and an inertial unit. It can operate a manipulator arm attached to its torso and has a wheeled base for moving around the room. Orbi-One includes a wireless access point, which allows WiFi communications with other robots and computers.

The software to control the robot hardware is based on a ROS framework. ROS is basically a set of libraries for robotics similar to operating system services, providing hardware abstraction for sensors and actuators, low-level device control, and inter-process communication. Computation takes place in processes named *Nodes*, which can receive and send *Messages*. Nodes publish Messages into information buffers called *Topics*.

### 2.3 KIO RTLS

In order to acquire ground-truth data about person location in the study area, we need an RTLS for indoor environments. The KIO RTLS commercial solution by Eliko^5^ has been used. KIO is a precise RTLS for tracking any object in 2- or 3-dimensional space. The Ultra Wideband technology enables to micro-position objects through obstructions. KIO also works in non-line-of-sight conditions and both indoors and outdoors.

KIO comes in two main configurations. The Regular Cell configuration guarantees a reliable accuracy of ±30 cm, according to the manufacturer’s specifications. The Small Cell configuration is designed for location-critical applications and provides reliable ±5 cm accuracy, according to the manufacturer’s specifications. Calibration done by the authors of this paper on the mock-up apartment shows that the error is higher in some areas, and lower in others, but on average, the claims of the manufacturer are correct.

KIO calculates the position of a mobile transceiver, called a *Tag*. In order to do so, KIO uses radio beacons, called *Anchors*, distributed in known positions in the surroundings. Figure 1D shows a KIO anchor. KIO tags are the same size and must be placed on-board the tracking subject, in our case people. The red dots in Figure 1C show the location of the six anchors used in these experiments. They are placed on the ceiling. The distribution of the anchors has been chosen following the method shown in Guerrero-Higueras et al. (2017).

### 2.4 Leg Detector (LD)

LD is a ROS package, which takes messages published by a LIDAR sensor as input and uses a machine-learning-trained classifier to detect groups of laser readings as possible legs. The code is available in a public repository,^6^ but is unsupported at this time.

LD publishes the location for the individual legs. It can also attempt to pair the legs and publish their average as an estimate of where the center of a person is. LD may optionally also publish visualization marker messages to indicate where detections happened.

### 2.5 PeTra

PeTra is a person-tracker tool for detecting and tracking, developed by the Robotics Group at the University of León. The system is based on a Convolutional Neural Network (CNN) using a configuration based on the U-Net architecture by Ronneberger et al. (2015).

The system performs the following steps in real time:

1. First, the data provided by the LIDAR sensor are processed to build a two dimensional occupancy map centered around the robot. This occupancy map is represented as a binary matrix, where 1s denote positions where the LIDAR scan found an obstacle, and 0s denote positions where the LIDAR scan either went through without detecting any obstacle or did not go through that position.
2. Then, the occupancy map is relayed to the network as input data. The network produces a second occupancy map representing the zones where legs have been detected.
3. Finally, center of mass calculations return the location of persons. PeTra also publishes locations for the individual legs and Marker messages for visualization.

### 2.6 Recording Procedure

**Figure 2:**
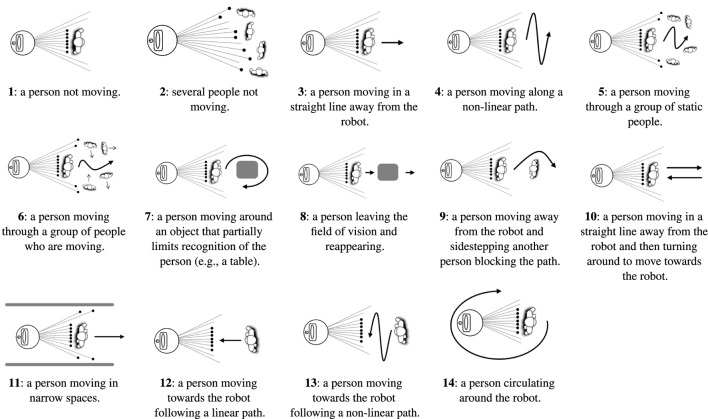
Recognition situations recorded.

The data were gathered in 14 different situations. In all of them, Orbi-One stood still as one or more people, carrying a KIO tag, moved around it. Three different locations for Orbi-One were defined (see Figure 1C) resulting in 42 scenarios (14 situations × 3 Orbi-One locations). Figure 2 shows the 14 different recognition scenarios recorded. These scenarios have been chosen according to different situations that may occur in robotics competitions such as ERL^7^ or RoboCup.^8^

A rosbag file^9^ was created for each scenario (except for situations 3, 12, and 13 where 3 rosbag files where recorded, for situation 4 where 4 rosbag files where recorded, and for situation 9 where 5 rosbag files where recorded), recording LIDAR sensor measurements, location estimates from PeTra and LD, locations from KIO RTLS, and other useful data. Specifically, the following data were included in the rosbag files:

- LIDAR sensor data. Data from LIDAR sensors are provided as ROS LaserScan Messages,^10^ which include, among other information, the following: acquisition time of the first ray in the scan, start/end angle of the scan, angular distance between measurements, and range data.
- PeTra location estimates provided as ROS PointStamped Messages,^11^ which include a position [*x, y, z*] and a timestamp.
- Location estimates calculated by LD. It publishes data for individual legs (as ROS PositionMeasurementArray Messages^12^). It also attempts to pair the legs and publishes their average as an estimate of where the center of a person is as a ROS PositionMeasurement Message.^13^
- Locations provided by KIO RTLS also provided as ROS PointStamped Messages.
- Messages from /*map*, /*odom*, and /*tf* ROS topics, which include map information, odometry of the robot base, and transformation information, respectively.

## 3 Results and Discussion

As a result of applying the recording method explained above, a first version of the dataset has been released. The dataset is known as “Range-based people tracker classifiers Benchmark Dataset”^14^ (RRID:SCR_015743). Data can be accessed at a public repository.^15^ Further information can be found at the University of Leon Robotics group web site,^16^ including information about contents of the rosbag files available for each scenario: start date/time, duration, and size.

The data gathered may be used to evaluate the performance of LD and PeTra. In order to empirically decide which one offers the best results, persons’ estimates from both systems can be compared to the ground-truth data provided by KIO RTLS. The accuracy error of PeTra (*e_PeTra_*) in a concrete instant of time can be calculated as the Euclidean distance between its location estimates (*l_PeTra_*) and ground-truth locations provided by KIO (*l_KIO_*). Equation (1) show *e_PeTra_* calculation, where *n* is the number of dimensions considered. In this case, only two dimensions need to be considered, since a mobile robot moves on the ground.

(1) $$e_{PeTra}=d(l_{PeTra},l_{KIO})=\sqrt{\sum_{i=1}^{n}\;(l_{PeTra_{i}}-l_{KIO_{i}})}.$$

The same can be done with the accuracy error of LD (*e_LD_*), calculated as the Euclidean distance between its location estimates (*l_LD_*) and ground-truth locations (*l_KIO_*) as shown in Equation (2).

(2) $$e_{LD}=d(l_{LD},l_{KIO})=\sqrt{\sum_{i=1}^{n}\;(l_{LD_{i}}-l_{KIO_{i}})}.$$

Once it is known how to calculate *e_PeTra_* and *e_LD_* in a concrete instance, it is possible to observe their evolution with time to select the system, which works better.

Regarding the above, there are two important issues to deal with:

1. KIO, PeTra, and LD use their own coordinate origins to represent locations. In order to compare these locations, they ought to be represented using the same coordinate origins. This can be done by using the translation and rotation quaternions published at the/tf topic.
2. Each message published for the recorded topics has its own timestamp with nanosecond precision, so, comparing locations in a concrete instant of time may not be an easy task. A synchronization method is needed to compare measurements from different topics. Pandas library, as seen in McKinney (2011), may be useful in doing so. It has merge methods to combine measurements by nearest timestamp.

Although the dataset was initially built to evaluate the performance of PeTra, it is important to note that any other people-tracker could be evaluated. Rosbag files include LIDAR sensor measurements, which may be used by any other people-tracker using LIDAR sensor measurements as input. By comparing location estimates with the ground-truth data provided by KIO, it is possible to calculate the accuracy error. In addition, these results can be compared with LD results, which are the most popular people-tracker using LIDAR sensor measurements.

## Author Contributions

PeTra is based on CÁ-A’s work for her end of degree assignment, which was proposed and supervised by VM. FM provided useful comments regarding the configuration of the neural network. MO carried out most of the data-gathering in León, supported by AG-H in León and FR-L in Luxembourg. AG-H and VM did the KIO calibration and drafted the manuscript.

## Conflict of Interest Statement

The authors declare that the research was conducted in the absence of any commercial or financial relationships that could be construed as a potential conflict of interest.

## Footnotes

^1^https://www.mrpt.org/robotics_datasets.

^2^http://robotica.unileon.es/index.php/Testbed.

^3^https://www.eu-robotics.net/robotics_league/erl-service/certified-test-beds/index.html.

^4^http://www.robotnik.es/manipuladores-roboticos-moviles/rb-one/.

^5^https://www.eliko.ee/products/kio-rtls/.

^6^http://wiki.ros.org/leg_detector.

^7^https://www.eu-robotics.net/robotics_league/.

^8^http://www.robocup.org/.

^9^A rosbag file is equivalent to a recording of the state of the robot in a period and can be used as dataset.

^10^http://docs.ros.org/api/sensor_msgs/html/msg/LaserScan.html.

^11^http://docs.ros.org/api/geometry_msgs/html/msg/PointStamped.html.

^12^http://docs.ros.org/jade/api/people_msgs/html/msg/PositionMeasurementArray.html.

^13^http://docs.ros.org/hydro/api/people_msgs/html/msg/PositionMeasurement.html.

^14^https://scicrunch.org/browse/resources/SCR_015743.

^15^https://figshare.com/articles/Benchmark_dataset_for_evaluation_of_range-based_people_tracker_classifiers_in_mobile_robots/5488744.

^16^http://robotica.unileon.es/index.php/Benchmark_dataset_for_evaluation_of_range-based_people_tracker_classifiers_in_mobile_robots#v1.0
